# Optimizing solar energy planning: A spatial zoning method based on characterized dimensionless radiation profiles

**DOI:** 10.1016/j.isci.2026.115545

**Published:** 2026-03-31

**Authors:** Yafei Wang, You Li, Chenhao Ren, Haolin Yang

**Affiliations:** 1 School of Civil Engineering and Architecture, Zhejiang University of Science and Technology, Hangzhou 310023, China; 2 Zhejiang International Science and Technology Cooperation Base for Low Carbon Building Material Technology with Recyclable Waste Resource, Hangzhou 310023, China; 3 Graduate School of Energy Science, Kyoto University, Yoshida-Honmachi, Sakyo-Ku, Kyoto 606-8501, Japan; 4 School of Management, Zhejiang University of Science and Technology, Hangzhou 310023, China; 5 Institute of Energy, Environment and Economy, Tsinghua University, Beijing 100084, China

**Keywords:** Energy resources, Energy sustainability, Energy management, Energy modeling

## Abstract

Spatiotemporal distribution of solar energy resources is crucial for overall energy development; however, most studies face challenges in achieving reliability and high accuracy when completing solar radiation datasets across regions and also assume fixed climate zones, overlooking randomness from regional climatic inconsistencies. This study proposed a method for identifying dimensionless characterized solar radiation profiles to fill in missing solar radiation values. Benefiting from the characteristic attributes contained in the dimensionless profiles, prejudgment based on multiple sets of profiles can provide a visual window for validating climatic consistency. A case study on Kyushu shows that the proposed method achieves high accuracy on untrained data, with R^2^ values of 0.92–0.93 daily and 0.89–0.90 hourly. A spatiotemporal solar radiation map of Kyushu was developed, showing average annual radiation of 4616–5320 MJ m^−2^ yr^−1^, and a weather-stochasticity-driven zoning approach that divides the region into four zones.

## Highlights

• A feature transformation method is proposed to remove daily geometric differences
• 16 dimensionless solar radiation profiles for assessing PV generation are created
• Prejudgment using various profile sets offers a visual check for local climate
• A precise, high-resolution model is achieved with an R^2^ of 0.92–0.93 in Kyushu

## Graphical abstract

**Figure:**
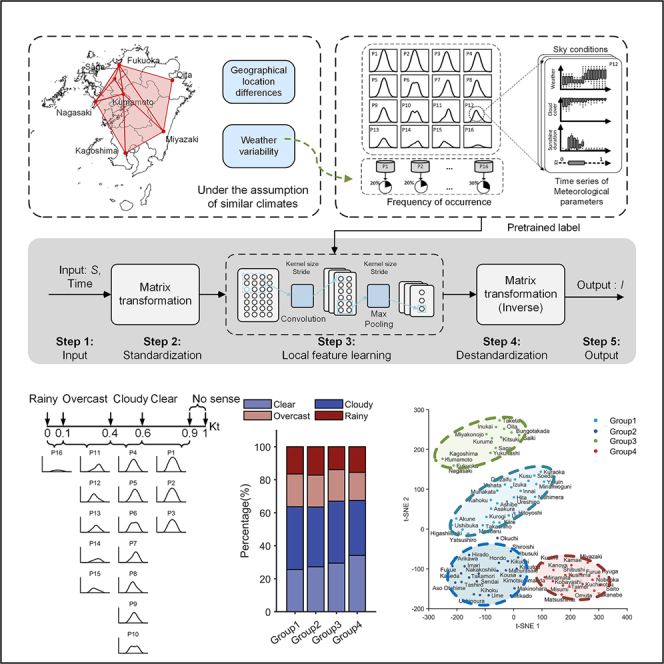

## Introduction

Solar energy is an environmentally friendly and clean renewable energy resource, and also one of the most accessible energy resources.^1^^,^^2^ Owing to the substantial drop in solar photovoltaic (PV) panel costs in recent years,^3^^,^^4^ PV technology is now regarded as a crucial method for addressing the global energy crisis and reaching carbon neutrality goals.^5^^,^^6^ Global solar radiation (I) is one of the critical factors determining the energy output of solar PV systems.^7^ The intensity and spatial distribution of global solar radiation are two key factors influencing solar energy development and optimal regional allocation.^8^ However, global solar radiation is not measured at most surface meteorological stations because of the high costs associated with instrumentation and maintenance. For example, only 48 official meteorological stations consistently gather global solar radiation data.^9^ Therefore, reconstructing comprehensive solar radiation datasets and deriving their spatial distribution from conventional meteorological variables is of considerable scientific and practical importance.

A detailed understanding of the spatial distribution of solar radiation directly benefits the design, deployment, and financial assessment of solar energy projects.^10^ Existing research generally infers missing solar radiation data using general meteorological parameters. Based on the input data, these methods can be categorized into satellite-based methods and surface parameter-based methods.

Satellite-based methods obtain large-scale, high spatial resolution information by observing the Earth’s surface from high altitudes.^11^^,^^12^ The key strength of this method is that it provides geographically continuous data that cannot be directly observed from the ground. Huang et al.^13^ developed a regionally adapted semi-empirical model based on Heliosat-2 to estimate global horizontal irradiance at the original resolution of the Fengyun-4 satellite (China’s latest-generation geostationary satellite series). Shi et al.^14^ estimated the global horizontal irradiance for China at a spatial-temporal resolution of 4 km and 15 min and subsequently created solar PV resource maps using the Fengyun-4A satellite data. However, due to interference from cloud cover, satellite-based methods tend to have relatively coarse temporal resolution, resulting in datasets that are often discontinuous or incomplete over time.^15^^,^^16^ Compared to the reanalysis data methods from discrete surface meteorological stations, satellite-based data methods do not demonstrate sufficient advantages in terms of accuracy, as they also face instability issues.^17^^,^^18^ On the contrary, studies have shown that reanalysis data methods from surface meteorological stations offer higher stability.^19^ Additionally, for most basic users, accessing these high-performance satellite products is quite challenging.^20^ These satellite products are usually very expensive. Therefore, although surface parameters may lack geographical resolution, they still provide a low-cost solution.

The method for generating solar radiation resource spatial distribution maps from surface meteorological stations generally involves three steps. First, establishing the nonlinear relationship between solar radiation and general meteorological parameters (including sunshine duration, air temperature, and relative humidity) at stations where radiation measurements are available. Then, solar radiation at general meteorological stations is estimated based on the established empirical relationship. Finally, using interpolation methods to fill in data for locations with no observations. Empirical models are favored in most studies due to their simplicity and interpretability. Angstrom^21^ proposed a simple linear equation that relates solar radiation to sunshine duration. Subsequent researchers^22^^,^^23^ have proposed various modified versions of this equation, including quadratic, cubic, and exponential forms. Jing et al.^24^ developed a generalized model (based on a cubic equation of sunshine duration) to obtain total solar radiation data and created spatial distribution maps for annual and seasonal solar radiation. Due to the unavailability of sunshine duration datasets and the need for higher accuracy, other studies have also incorporated temperature and humidity to construct their models.^25^^,^^26^

However, while sufficient for resource assessment, most of these studies focus on calculating daily values or monthly average daily values. The more complex nonlinear relationship between hourly solar radiation and meteorological parameters makes these classic models less applicable. Currently, the most accurate hourly models are the Zhang and Huang model^27^ and its modified versions.^28^ Meanwhile, Machine learning models have advantages in handling these high-dimensional nonlinear relationships.^29^^,^^30^ Numerous studies^31^^,^^32^ have summarized the advantages of various machine learning techniques in solar radiation forecasting and the resource assessments generated based on these methods (artificial neural network (ANN),^33^ support vector machine (SVM),^34^ convolutional neural network (CNN), long-short term memory (LSTM)^35^ and their combinations^36^). Due to the lower computational cost, machine learning-based methods typically incorporate many surface parameters (including atmospheric pressure, ambient temperature, relative humidity, precipitation, wind speed, dew point temperature, and sunshine duration) to increase accuracy. However, the mechanisms of solar radiation transmission remain unclear, making it difficult for ML models to explain the autocorrelation and interaction effects among multiple input parameters.^37^ This issue is amplified in multi-point predictions, as the models need to be recalibrated.

Additionally, despite their advantages in accuracy, these methods often overlook an important issue during the process of completing datasets: validating the climatic consistency of the experimental region. It is well-known that empirical models require coefficient calibration before application. Similarly, in ML models, hyperparameter optimization is typically needed to achieve higher performance. Therefore, the common approach is to divide different regions and establish separate empirical or ML models for each.^24^^,^^38^ Different model coefficients, hyperparameters, and structures can lead to significant variations in the results. Current solutions are mostly based on prior knowledge (using climatic zones for pre-classified modeling) and do not adjust the models for the regional differences in solar radiation itself.

The solar radiation profile refers to the distribution of solar radiation throughout a day. This includes the variation in solar radiation intensity over time, from sunrise to sunset. Similar to some feature engineering (such as representative load profiles in building simulation^39^^,^^40^), identifying a set of representative solar radiation profiles to describe the climatic conditions related to solar radiation at a particular location is an intuitive solution. However, due to the seasonal variations in solar radiation and the geographical differences across multiple observation points, the geometric differences exhibited by solar radiation profiles are significant (specifically in terms of sunrise and sunset times, as well as the overall intensity of solar radiation). It is challenging to cluster into a set of representative feature profiles. Therefore, we aim to propose a concept: dimensionless solar radiation profiles. A method for describing solar radiation profile shapes that removes seasonal and geographical differences. Additionally, the proposed dimensionless profiles can be correlated with time series of other meteorological parameters to predict hourly solar radiation. Thanks to the differences in profile shapes, we can first decide whether to apply the same set of radiation profiles by visually comparing representative radiation profiles from different locations before making multi-point predictions. This offers a promising solution to the issue of validating climatic consistency mentioned earlier.

Therefore, it is crucial to consider how to deseasonalize a solar radiation time series to obtain dimensionless profiles. In light of the preceding studies, this paper aims to address research gaps as follows:

Issue 1: Robust physical models are often not capable of accommodating the inherent uncertainties of complex weather conditions, leading to insufficient prediction resolution and accuracy. Meanwhile, a lot of deep learning models are treated as black-box approaches, making it difficult to interpret the interaction effects among multiple input parameters and lacking fundamental trustworthiness. There is a lack of a framework that balances simplicity, reliability, and high accuracy in the task of completing solar radiation datasets across multiple locations.

Issue 2: Most studies address the issue of climatic consistency from different experimental regions based on prior knowledge (assuming regional modeling based on existing climate zones). Once models are established (including coefficients and hyperparameters), they are difficult to change without being considered new models. There is a lack of visualization methods to validate this assumption, thus overlooking the risk of randomness due to regional climatic inconsistencies.

Responding to the limitations discussed above, this study provides the following contributions.

• A transformation-matrix-based feature mapping approach was developed to eliminate the inherent geographical and seasonal differences in solar radiation, providing the capability to the construction of dimensionless, characteristic solar radiation profiles that capture its fundamental variability.
• The dimensionless profiles provided a clear understanding of the correlation between input parameters and solar radiation. Therefore, selecting a single meteorological parameter (sunshine duration) as the input can also yield high-accuracy and easily interpretable results.
• A set of dimensionless profiles can be regarded as representative features of the experimental region. Therefore, prejudgment based on multiple sets of profiles can provide a visual window for validating climatic consistency, thereby enhancing the model’s applicability across multiple locations.

## Results and discussion

### Variability in multi-observation

The variability in data distribution across multiple observation points affects the ultimate efficacy of the model. Therefore, it is essential to first ascertain the differences and correlations among the solar radiation datasets collected from the seven observation points (They are Fukuoka (FUK), Oita (OIT), Nagasaki (NAG), Saga (SAG), Kumamoto (KUM), Miyazaki (MIY), and Kagoshima (KAG).). Given that the seven observation points are evenly distributed across Kyushu Island and are relatively distant from each other, the temporal information contained within the original data complicates comparisons of the core factors (such as the randomness of weather) that influence variations in solar radiation at these points. Initially, the data from the seven observation points is normalized through matrix transformation to remove the temporal information in the raw data caused by geographical location and changes in solar elevation, using the Tokyo station as the standard coordinate system.

**Figure 1:**
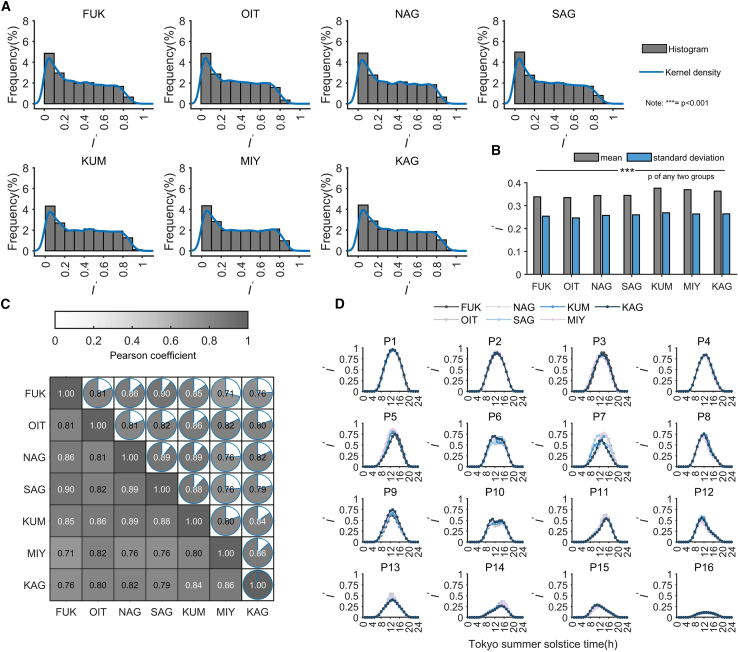
Variability in the solar radiation datasets from the seven observation points on Kyushu Island from 2000 to 2021 (A) Frequency distribution. (B) Mean, standard deviation, and significance testing of the overall dataset. (C) Pairwise correlation coefficients between groups. (D) Sixteen representative feature types.

Figure 1A displays the histogram of the normalized data distribution for the seven observation points over a 22-year period from 2000 to 2021. The blue line represents the estimated kernel density distribution curve. It can be observed that the seven observation points share a similar distribution form. Specifically, the proportion of low radiation levels (I'<0.1) is the highest at approximately 5%, the range of 0.2 < I'<0.8 is nearly uniform, with each interval constituting close to 2%, and values greater than 0.8 comprise only a small portion. Figure 1B presents the mean and standard deviation for the combined datasets from the seven observation points. The results indicate that the average values for the datasets from FUK, OIT, NAG, and SAG are similar, approximately 0.35. In contrast, the average value for the datasets from KUM, MIY, and KAG is higher, approximately 0.38. Figure 1C displays the pairwise correlations among the seven combined datasets, showing that any two observation points exhibit good correlation (correlation coefficients ranging from 0.71 to 0.90). Additionally, significance tests were conducted for any two sets of experiments, and the results indicate that the *p*-values for any two sets are less than 0.05.

Using the K-Means++ clustering method, feature extraction was performed on the datasets from the seven observation points, resulting in 16 distinct feature types. These are displayed in Figure 1D. Except for the P7 location in KAG, the feature types at the seven sites conform to our basic assumption regarding the 16 types of skewed distributions, which include three clear day types (P1, P2, P3), four clear-to- overcast day types (P4, P8, P12, P15), four overcast-to-clear day types (P5, P7, P11, P14), three overcast day types (P9, P13, P16), and two clear interval overcast day types (P6, P10). For each representative type among the seven observation points, pairwise combinations can generate 21 combination scenarios. The correlation tests between these combinations are displayed in the supplemental information (Figure S1). Blue dots represent one of these combination scenarios, totaling 21 points. The results indicate that any two observation points exhibit significant correlation across the 16 representative feature curves. Therefore, after comparing and analyzing the measured datasets and data distributions from the seven observation points on Kyushu Island, we can conclude that the solar radiation across Kyushu Island, with temporal information removed, possesses similar characteristic features.

### Feature curves and accuracy analysis

Due to the lack of actual solar radiation data at over 70 other weather stations on Kyushu Island, FUK, NAG, KUM, MIY, and KAG are selected from the seven stations with actual measurements as sites for generating feature curves and modeling. OIT and SAG were chosen as the unlabeled datasets for accuracy validation.

**Figure 2:**
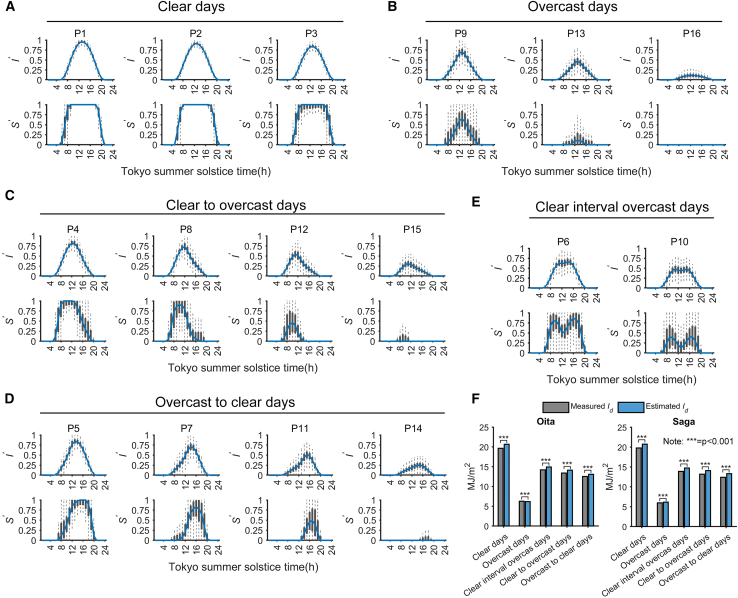
Basic feature assumptions and model accuracy validation (A) Clear days. (B) Overcast days. (C) Clear-to-overcast days. (D) Overcast-to-clear days. (E) Clear-interval-overcast days. (F) Comparison of average predicted values under different weather conditions and significance testing.

The sixteen feature types for Kyushu Island, developed from the aforementioned five sites, are displayed in Figures 2A–2E (To better compare differences and enhance generalizability, Tokyo station is selected as the standard Cartesian coordinate.). The black bar graph represents the boxplots derived from the original datasets of each category, while the blue line indicates the representative curve for each type. It can be observed that the representative curves generated from the five locations conform to the basic assumptions about the 16 types of skewed distributions that are established in our previous research (which focused on Tokyo, supplemental information (Figure S2). These include three clear day types (Figure 2A), four clear-to- overcast day types (Figure 2C), four overcast-to-clear day types (Figure 2D), three overcast day types (Figure 2B), and two clear interval overcast day types (Figure 2E). Additionally, Figures 2A–2E also display the correlation between the environmental parameter used for input (sunshine duration) and solar radiation. From this, it can be observed that the sunshine duration, after matrix transformation, exhibits good temporal consistency with the solar radiation values. Therefore, accuracy validation for OIT and SAG is conducted using the feature curves generated from the five locations.

Model validation is divided into two scales: first, using daily cumulative values as the basic unit for resource assessment, and second, deeper validation at the hourly level. The validation period spans 22 years, covering 8,036 consecutive natural days from 2000 to 2021. Figure 2F shows the daily value averages by weather type and significance validation. The results indicate that despite some discrepancies in the predictions (manifested as differences in average values), the model is effective and significant under various weather conditions. Additionally, most solar radiation resource assessment studies are conducted at daily resolution. However, in our research, daily values are represented in the form of feature curves, making the generation of finer-grained data one of the strengths of this study.

**Figure 3:**
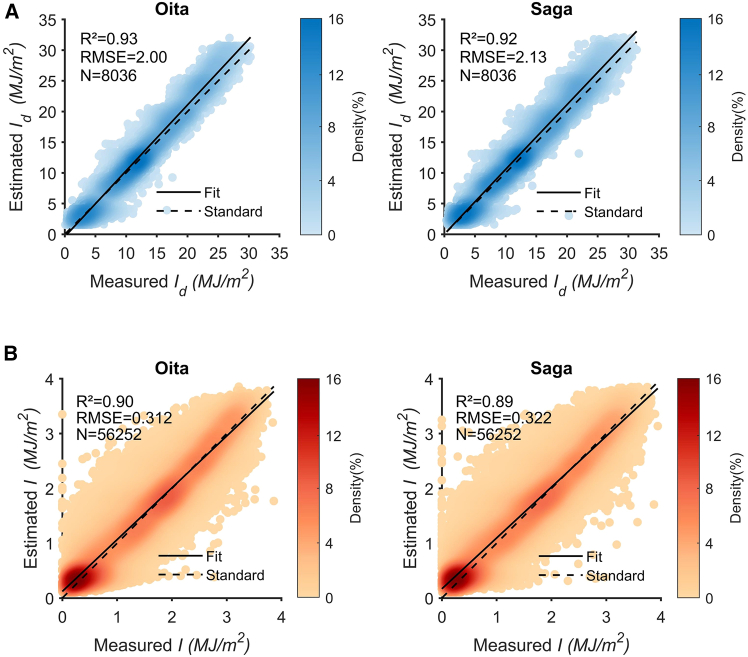
Scatterplot of calculated versus measured daily and hourly solar radiation values (A) Daily. (B) Hourly. (Note: Data from 0:00 to 7:00, as well as from 17:00 to 23:00, are not included for correlation analysis to prevent an overestimation of model accuracy at low solar altitude angles. These changes do not affect the conclusions.).

Figure 3A displays the scatterplots of daily values (Id) for two locations. The long-term validation at OIT and SAG demonstrates the effectiveness of the model, with R-squared values of 0.93 and 0.92, respectively. The RMSE values are 2.0 MJ m^−2^ d^−1^ and 2.13 MJ m^−2^ d^−1^. Similar studies (Jing et al., 2023) using traditional methods (point-to-point fitting) show that the accuracy of daily values ranges from 0.89 to 0.90, with RMSE values between 2.2 MJ m^−2^ d^−1^ and 2.37 MJ m^−2^ d^−1^. Compared to these studies, the proposed method achieves higher accuracy. Figure 3B displays the scatterplots of hourly values for two locations. The long-term validation at OIT and SAG confirms the effectiveness of the model, with R-squared values of 0.9 and 0.89, respectively. The RMSE values are 0.312 MJ m^−2^ and 0.322 MJ m^−2^, demonstrating higher performance at finer granularity. Additional statistical metrics can be found in Table 1. The parameters used are from Table 2. A noticeable dispersion is observed at both sites, where the data deviates from the standard curve (y = x). This deviation stems from the use of representative profiles that reflect high probability expected values, leading to the normalization and averaging of certain extreme cases. Future research will aim to mitigate this limitation by integrating deep learning approaches to better capture non-linear variations. Nevertheless, we note that the deviations constitute only a small fraction of the data, which is acceptable in resource assessment and feature analysis contexts. Moreover, Further discussion on model transferability and adaptation across fundamentally different climatic regions, such as tropical and arid zones, can be found in studies^41^^,^^42^ and will be systematically explored in our future work.

**Table 1:** Evaluation of statistical metrics (all data were analyzed by the validation dataset from January 1, 2022, to December 31, 2022)

Resolution	Site	R2	RMSE (MJ m^−2^)	rRMSE (%)	MSE (MJ m^−2^)	Nash–Sutcliffe Equation (NSE)
Daily	Oita	0.93	2.00	14.85	4.00	0.937
	Saga	0.92	2.13	16.00	4.55	0.928
Hourly	Oita	0.90	0.31	22.75	0.13	0.863
	Saga	0.89	0.32	23.94	0.13	0.853

**Table 2:** Parameters recorded at Kyushu observatories

Parameters	Latitude	Longitude	Time	Sunshine duration	Global solar radiation
Symbol	*–*	*–*	*t*	*S*	*I*
Standardized	*–*	*–*	*–*	*S’*	*I’*
Unit	degree	degree	*–*	hour	MJ m^-2^

### Spatial distribution of global solar radiation

**Figure 4:**
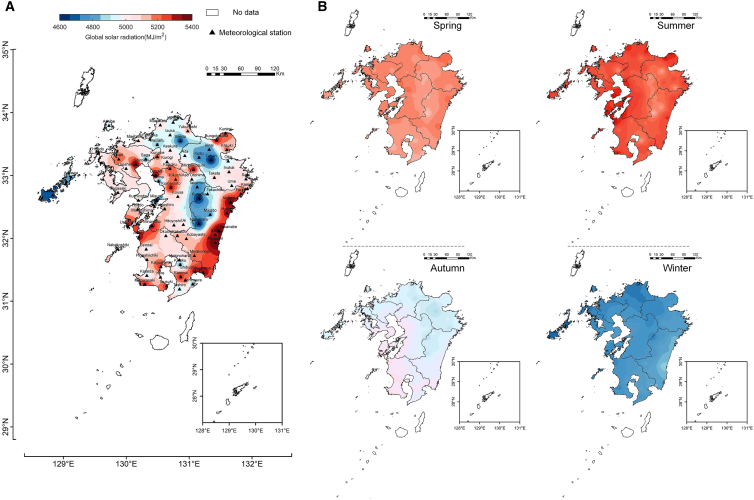
Spatial distribution and temporal evolution of global solar radiation in Kyushu (A) Annual average global radiation from 2000 to 2021. (B) Seasonal average global radiation from 2000 to 2021.

The graphical decomposition model predicts hourly level solar radiation for the remaining 75 observation points on Kyushu Island from 2000 to 2021, serving as the foundational data for resource assessment. Data for other regions without meteorological observations is interpolated using the inverse distance weighting method. Additionally, because the climate and weather conditions of islands may differ from those of the mainland, remote islands (such as Okinawa) are not included in the scope of this study. Figure 4A shows the average annual solar radiation during a 22-year observation period on Kyushu Island. The results indicate that Kyushu’s average annual solar radiation ranges between 4616 and 5320 MJ m^−2^ yr^−1^, exhibiting stronger intensities in the southeastern as well as southwestern regions, while the central and northern regions exhibit comparatively lower levels. The primary reason for this variation in Kyushu’s solar radiation is the concentration of mountain terrain in the central regions, particularly around the Aso and Kunimi. The mountain ranges impede maritime airflows from the surrounding seas, intensifying local evaporation and contributing to higher atmospheric turbidity. Additionally, the study shows that the areas receiving the highest solar radiation are mainly concentrated along the coastal regions of Akune, Minamata, MIY, and Nobeoka, where the annual totals range from 5200 to 5400 MJ m^−2^ yr^−1^.

In Kyushu, spring spans from March to May, summer from June to August, autumn from September to November, and winter from December to February according to the climate. Figure 4B shows the seasonal total radiation for Kyushu. The graph illustrates that the seasonal distribution varies compared to the annual data. During the summer, the total solar radiation reaches its maximum in the southwestern coastal regions, particularly around Akune and Minamata, with values ranging from 1700 to 1732 MJ m^−2^. In the southeastern coastal regions of MIY, Saito, and Hyuga, the total radiation resources during the summer are approximately 1500–1525 MJ m^−2^. In winter, the scenario reverses; the total solar radiation in the southwestern coastal regions of Akune and Minamata, is near its lowest, ranging from 720 to 750 MJ m^−2^. Conversely, in the southeastern coastal regions of MIY, Saito, and Hyuga, the total radiation resources in winter are the highest, approximately 914–978 MJ m^−2^. Therefore, although the total annual solar energy resources on the east and west coasts are similar, the seasonal distribution varies significantly. It can be summarized that the eastern coast exhibits relatively small seasonal fluctuations between summer and winter, whereas the western coast experiences more pronounced seasonal variations.

**Figure 5:**
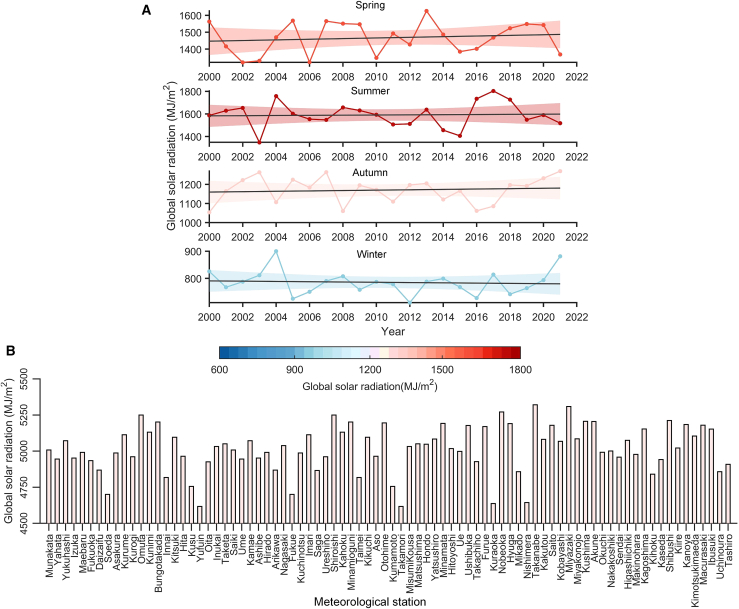
Temporal variation of global solar radiation in the Kyushu region (A) Temporal evolution of global solar radiation in Kyushu. (B) Annual average global radiation of 82 meteorological stations.

The temporal evolution of Kyushu’s seasonal total solar radiation is presented in Figure 5A. In Kyushu, the average total solar radiation resources for spring, summer, autumn, and winter are approximately 1467, 1590, 1170, and 785 MJ m^−2^, respectively. Figure 5A also provides the fitted lines for total radiation over time for each season. The results indicate that despite significant annual and seasonal variations in solar radiation, there is a long-term increasing trend in spring, summer, and autumn, while winter shows a decreasing trend in total radiation. The rates of increase are approximately 2%, 1%, 1.8%, and −2.7%, respectively. Additionally, the annual average total radiation for each meteorological observation point is shown in Figure 5B.

### Solar radiation zoning driven by weather stochasticity

Studies on solar radiation zoning are not uncommon. Most research^43^^,^^44^ classifies the spatial variation of solar radiation in a region into several zones based on average air temperature (including minimum and maximum air temperatures), relative humidity, sunshine duration, and global solar radiation. These studies deepen the understanding of the external characterization parameters of solar radiation in different regions (such as temperature variation), and they directly aid engineering applications. However, there are still challenges in explaining the intrinsic factors that drive regional variations in solar radiation. In our hypothesis, the factors influencing regional variations in solar radiation can be summarized as follows: 1. Geographic and temporal elements: Theoretically, it is well understood that the closer to the equator, the richer the solar radiation resources. These determine the upper limit of the total radiation. 2. Randomness of weather changes: Murky air determines the lower limit of the total radiation. Therefore, the randomness of weather can be considered a key parameter influencing regional radiation variations. In our study, the murkiness of the weather based on radiation is quantified into 16 feature types. These types are dimensionless, allowing for comparisons throughout the year.

**Figure 6:**
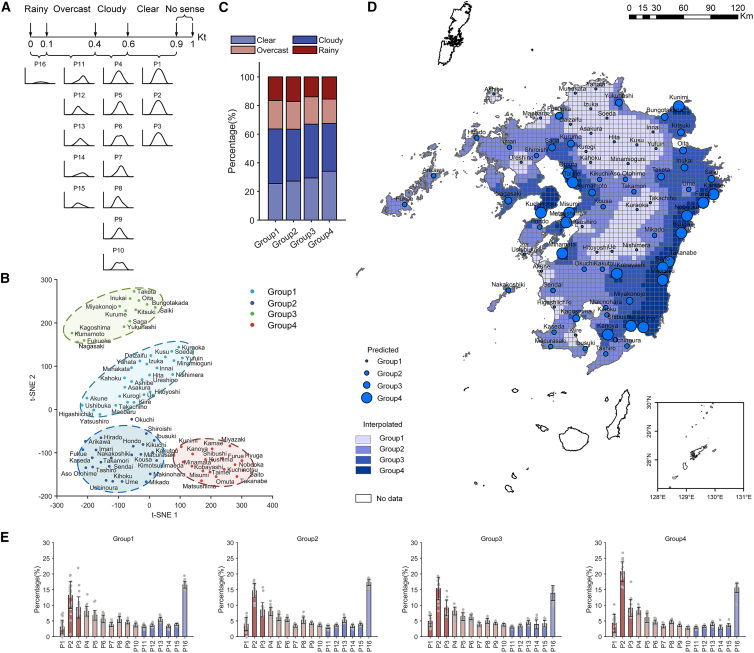
Solar radiation zones driven by weather stochasticity (A) Correspondence between the sixteen profiles and Kt. (B) Dimensionality reduction and visualization of the zoning using t-SNE. (C) Weather occurrence probabilities for each of the four groups. (D) Zoning map in Kyushu driven by weather stochasticity. (E) Occurrence probabilities of the sixteen profiles within each of the four groups.

Figure 6A illustrates the conversion relationship between these 16 types and the clearness index (Kt). Typically, the clearness index (Kt) categorizes the weather conditions throughout the day into three or four classes. According to the magnitude of Kt, the sky conditions can be roughly classified as clear day (Kt ≥ 0.6), cloudy (0.4 ≤ Kt < 0.6), overcast day (0.1 ≤ Kt < 0.4), and rainy (Kt < 0.1). Based on these four types of sky murkiness (clear, cloudy, overcast, rainy), the intrinsic factors causing regional variations in solar radiation can be explored and zoned. t-SNE is used to deconstruct the potential data structure based on the four types of sky murkiness over a 22-year period and to distinguish the solar radiation regions in Kyushu. Figure 6B shows the dimensionality reduction visualization results of sky murkiness conditions in the Kyushu region using the t-SNE method. The axes of the t-SNE plot do not have any interpretable meaning.^45^ The results show that the high-dimensional data clusters in the two-dimensional coordinate system generated by t-SNE form four distinct groups. Therefore, Kyushu can be divided into four distinct regions based on weather heterogeneity. The groups are labeled and assigned unique colors for better visualization.

Figure 6C presents the average characteristics and distribution proportions of the four types. The proportions of the four types of sky murkiness in Group 1 are 25.5%, 38.1%, 19.8%, and 16.5%, respectively. In Group 2, the proportions are 27.2%, 36.3%, 19.3%, and 17.2%, respectively. Group 3 has proportions of 29.4%, 37.4%, 19.1%, and 13.9%, respectively. Finally, in Group 4, the proportions are 34.1%, 33.4%, 16.9%, and 15.5%, respectively. It can be observed that the overcast and rainy types exhibit smaller variations in their cumulative occurrence probabilities (with a maximum change of 2.9–3.3%), whereas the clear type shows the greatest variation among these groups. Specifically, compared to Group 1, the cumulative occurrence probability of clear skies in Group 4 has increased by 8.6% over the years. Figure 6D shows the spatial distribution of the four groups across Kyushu Island. The grid resolution is 5 km × 5 km. The results reveal an interesting pattern: The zones displayed almost perfectly match the regional distribution trends of the annual average total solar radiation shown in Figure 6A. This manifests as the Group 4 regions, dominated by clear skies, being primarily distributed in the southwestern coastal regions, including Akune and Minamata, as well as the southeastern coastal regions, including MIY, Saito, and Hyuga. The Group 1 regions, dominated by overcast and rainy conditions, are primarily concentrated in the northern and central mountainous areas such as Soeda, Hita, Ue, and Kuraoka. Generally, geographic and temporal factors are decisive in influencing the total amount of solar radiation. However, Figure 6A does not show a clear north-south difference; instead, it closely aligns with the regional distribution based on weather stochasticity. Therefore, at the geographical scale of this study, weather stochasticity and the proportions of various weather types are key factors influencing the total amount of solar radiation.

### Limitations of the study

**Figure 7:**
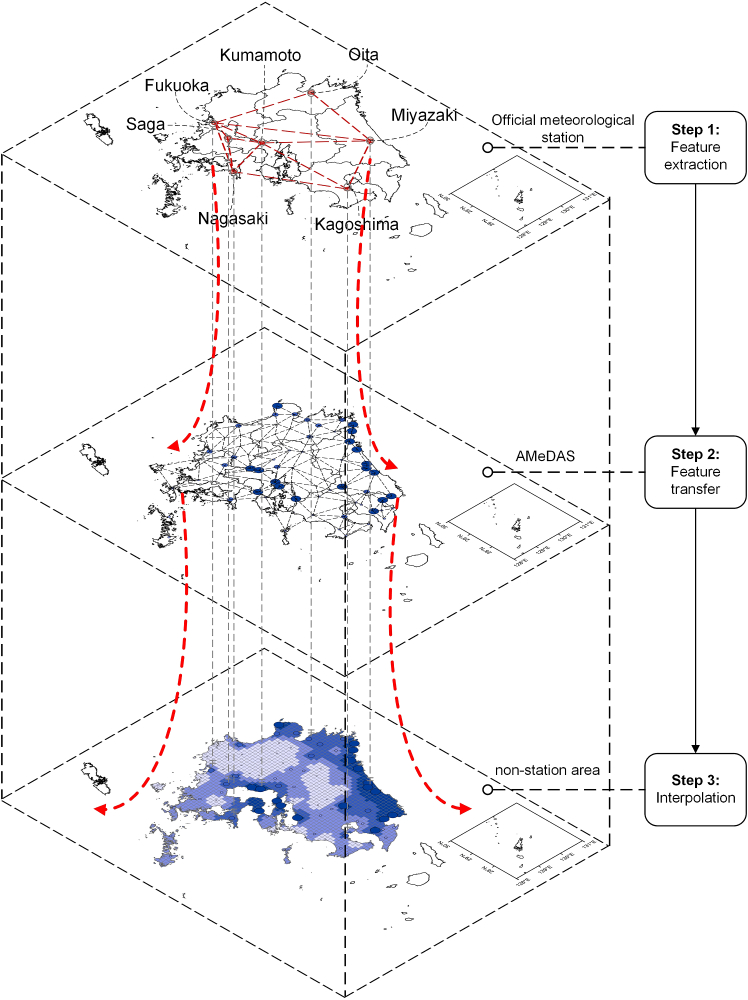
Main idea of feature extraction and transfer

**Figure 8:**
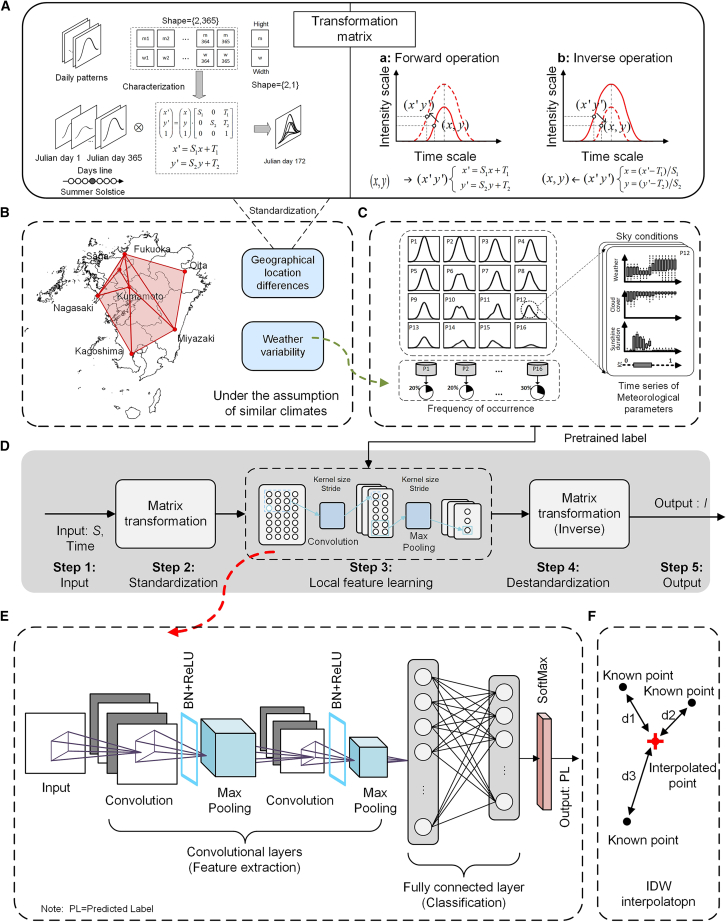
Overview of the process of the proposed method (A) Deseasonalization and dimensionless method based on the transformation matrix. (B) Locations of the seven official meteorological stations. (C) Feature extraction using K-means. (D) Overall estimation process. (E) Classification task using CNN. (F) Inverse distance weighing interpolation.

**Figure 9:**
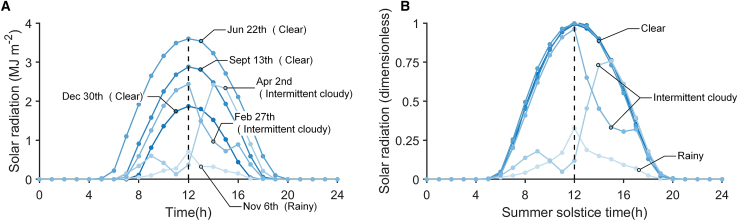
Seasonal differences in solar radiation data (A) Raw. (B) Transferred.

Despite our proposed method (The workflow is based on Figures 7, 8, and 9) providing a potential means for validating climatic consistency, the actual calculation process still carries a risk of randomness in climate assessment for sites lacking measured solar radiation data. Acknowledging these limitations provides a stepping stone for future research. We propose a solution to this issue: supplementing the climate-suspect sites with intermittent measured solar radiation data to determine the climatic conditions at those points. The specific observation periods and minimum time lengths involved need to balance the required granularity and economic feasibility, which we will explore further in future studies.

## Resource availability

### Lead contact

Requests for further information and resources should be directed to and will be fulfilled by the lead contact, You Li (liyou.rs@outlook.com).

### Materials availability

This study did not generate new unique materials, cell lines, or biological resources.

### Data and code availability

• All data were downloaded from the Japan Meteorological Agency (JMA) and are accessible at https://www.data.jma.go.jp/risk/obsdl/index.php. The rationale for using these datasets is discussed in the STAR Methods. All data reported in this article are also available in the supplemental information.
• This article does not report any original code.
• Any additional information required to reanalyze the data reported in this paper is available from the lead contact upon request.

## Acknowledgments

This work was supported by the project from the 10.13039/501100008990Department of Science and Technology of Zhejiang Province under grant no. 2026C04035, and by the 10.13039/501100001691Japan Society for the Promotion of Science (10.13039/501100001691JSPS) under grant nos. P25418 and 25KF0233. The authors would also like to acknowledge the collection and collation of weather data by the 10.13039/501100023210Japan Meteorological Agency (https://www.jma.go.jp/jma/index.html).

## Author contributions

Conceptualization, Y.W. and Y.L.; methodology, Y.W. and Y.L.; writing – original draft, Y.W.; writing – review and editing, C.R. and H.Y.; funding acquisition, Y.L. and Y.W.; resources, Y.L. and Y.W.; supervision, Y.L.

## Declaration of interests

The authors declare no competing interests.

## STAR★Methods

### Key resources table

**Table:** 

REAGENT or RESOURCE	SOURCE	IDENTIFIER
**Software and algorithms**		

Windows 11	Microsoft	https://www.microsoft.com/windows/
Matlab (R2024a)	MATLAB	https://www.mathworks.com/products/new_products/release2024a.html

**Other**		

Weather Data Resource	Japan Meteorological Agency	https://www.jma.go.jp/jma/index.html

### Method details

The method for generating solar radiation resource spatial distribution maps from surface meteorological stations generally involves the following steps. First, establishing the nonlinear relationship between solar radiation and general meteorological variables (including sunshine duration, relative humidity, as well as air temperature) based on data from stations equipped with solar radiation sensors. Second, this relationship is applied to estimate solar radiation at general meteorological stations. Then, using interpolation methods to fill in data for locations with no observations. Finally, regional solar radiation is assessed using the generated spatiotemporal distribution maps.

Since the main contribution of this paper is the introduction of a new method based on dimensionless profiles, Figure 7 shows the main idea of feature extraction and transfer and Figure 8 shows the workflow, and the details of this method will be elaborated in the following section:

The workflow (Figure 7) can be detailed as:

Data standardization: The extraterrestrial solar radiation profile for the measured location is calculated using the time and geographical parameters (latitude and longitude). Then, the measured solar radiation or meteorological parameter profiles (24-hour) are projected onto a selected standard coordinate axis using a transformation matrix (Figure 8A).

Feature extraction: Representative solar radiation and meteorological parameter profiles are extracted using the unified coordinate axis. The profiles are generated using the K-means++ clustering algorithm, while pre-trained labels were generated based on solar radiation distribution patterns observed over a 22-year period, between Jan. 1, 2000, and Dec. 31, 2021 (Figure 8C).

Feature transfer: By leveraging the correlation between meteorological parameters and solar radiation, feature transfer is converted into a classification task. Representative profiles for locations without observation stations are then calculated using the input meteorological data. This stage includes training the CNN model through datasets between Jan. 1, 2000, and Dec. 31, 2021 (Figure 8E).

Data destandardization: The scaled radiation patterns are inversely transformed to recover their original scale, corresponding to the actual temporal measurements at the test sites. At the same time, the model generates the anticipated solar radiation values (Figures 8A and 8B).

#### Deseasonalization

Seasonal variations in solar radiation are governed by systematic geometric factors; simple normalization is insufficient to achieve comparability across dates. Therefore, a matrix-based coordinate transformation is employed to simultaneously adjust the temporal and amplitude dimensions of daily radiation profiles, allowing radiation patterns from different seasons to be represented within a unified, dimensionless framework. Figure 9 shows a representative example of the transformation process and its resulting effect.

From a two-dimensional perspective (Flattening the solar radiation data onto a 24-hour time coordinate axis.), the geometric discrepancies among curves with similar solar radiation patterns are primarily attributed to inconsistencies in coordinate scaling. This limitation can be effectively resolved using transformation matrices, which enable unified operations such as scaling, rotation, and translation. The scaling of solar radiation mainly involves 2D variations in time and intensity; a 2D linear transformation is therefore employed, as formulated in Equation 1:

(Equation 1) $$\left[\begin{array}{cc} a_{11} & a_{12}\\ a_{21} & a_{22} \end{array}\right]\times \left[\begin{array}{l} x\\ y \end{array}\right]=\left[\begin{array}{l} a_{11}x+a_{12}y\\ a_{21}x+a_{22}y \end{array}\right]$$

A transformation matrix converts one vector into another vector, providing a geometric interpretation applicable in both 2D and 3D spaces. In this research, shearing and scaling transformations are applied to normalize the temporal and intensity dimensions of solar radiation data.

a. Shearing along the y-axis:

(Equation 2) $$\text{shear}-y\left(\text{dx},\text{dz}\right)=\left[\begin{array}{ccc} 1 & 0 & \text{dx}\\ 0 & 1 & \text{dz}\\ 0 & 0 & 1 \end{array}\right]$$

b. Scaling:

(Equation 3) $$\text{scale}\left(\text{Sx},\text{Sy},\text{Sz}\right)=\left[\begin{array}{ccc} \text{Sx} & 0 & 0\\ 0 & \text{Sy} & 0\\ 0 & 0 & \text{Sz} \end{array}\right]$$

The procedure for computing the daily extraterrestrial solar radiation profile at a given longitude, latitude, and time based on the solar altitude angle is detailed in Whiteman and Allwine.^46^ The extraterrestrial solar radiation profile is assumed to follow an ideal bell-shaped (Gaussian) distribution, and its geometric characteristics can therefore be described as Equation 4:

(Equation 4) $$\xi \left(d\right)=\rho_{d}\times \exp-{\left(\frac{t-\upsilon_{d}}{\omega_{d}}\right)}^{2}$$

where *ρ*_*d*_, *ω*_*d*_ and *ʋ*_*d*_ represent key features of the Gaussian curve of the extraterrestrial solar radiation profile, representing the maximum value, width and offset value, respectively. *ρ*_*d*_ represents the peak value of the daily solar radiation distribution curve, *ω*_*d*_ denotes the effective day length of the daily solar radiation distribution curve, and *ʋ*_*d*_ describes the offset between local time t and solar time. Since the periodic variation of solar radiation is symmetrically distributed around the summer solstice, the transformation matrix can be described as the ratio of the feature parameters to the period of the summer solstice.

(Equation 5) $$F\left(d,t\right)=\left(\begin{array}{l} x^{\prime }\left(t\right)\\ y^{\prime }\left(t\right)\\ 1 \end{array}\right)=\left(\begin{array}{l} x\left(t\right)\\ y\left(t\right)\\ 1 \end{array}\right)\times \left[\begin{array}{ccc} \frac{\omega_{0}}{\omega_{d}} & 0 & 12\times \left(1-\frac{\omega_{0}}{\omega_{d}}\right)\\ 0 & \frac{\rho_{0}}{\rho_{d}} & 0\\ 0 & 0 & 1 \end{array}\right]$$

here d denotes the day index, defined within the set D = {1, 2, …, 365}, and t denotes the hourly index, defined within T = {1, 2, …, 24}. The parameters *ρ*_*d*_ and *ω*_*d*_ characterize the Gaussian-shaped daily extraterrestrial solar radiation curve, corresponding to its peak magnitude and curve width, respectively, and *ρ*_*0*_ and *ω*_*0*_ indicate these reference values on the summer solstice. x'(t) and y'(t) represent the transformed coordinates obtained from the original coordinates x(t) and y(t). Moreover, because the dimensionless input parameter (sunshine duration) remains invariant in terms of seasonal intensity, it only requires maintaining its temporal correspondence with solar radiation, as formulated in Equation 6:

(Equation 6) $$G\left(d,t\right)=\left(\begin{array}{l} x^{\prime }\left(t\right)\\ y^{\prime }\left(t\right)\\ 1 \end{array}\right)=\left(\begin{array}{l} x\left(t\right)\\ y\left(t\right)\\ 1 \end{array}\right)\times \left[\begin{array}{ccc} 1 & 0 & 12\times \left(1-\frac{\omega_{0}}{\omega_{d}}\right)\\ 0 & 1 & 0\\ 0 & 0 & 1 \end{array}\right]$$

For a comprehensive description of the transformation matrix and its physical interpretation, please refer to the Li et al.^41^

#### Feature extraction

Dimensionless solar radiation provides a basis for comparing radiation distribution states across different geographic locations and seasons. Consequently, general unsupervised methods can be used to cluster and mine the intrinsic features of the data. In this work, the K-means++ algorithm is adopted to enhance clustering stability and reduce sensitivity to local minima, which commonly arise from the random initialization of cluster centers.^47^ Unlike the conventional K-means method that randomly selects K initial centroids from the dataset, K-means++ initializes K cluster centers with maximal mutual separation, thereby improving the robustness and consistency of the clustering process.

The method is outlined as follows:

a. A single sample is randomly selected from the dataset to serve as the initial cluster center, denoted as *c*_*1*_*.*
b. For each data point, compute its minimum distance to the existing cluster centers (i.e., the distance to the nearest cluster center), denoted as *D(x).* Next, compute the selection probability *p(x)*, which determines the likelihood of each sample being chosen as the next center.

(Equation 7) $$p\left(x\right)=\frac{D{\left(x\right)}^{2}}{\sum_{xÎX}D{\left(x\right)}^{2}}$$

Select the next cluster center using the roulette wheel selection method.

c. Duplicate step (b) till a total of *K* cluster centers is selected.
d. For every sample x in the dataset, compute its distance to the *K* cluster centers and assign it to the class corresponding to the cluster center with the minimum distance.
e. For each class *c*, recompute the cluster center.

(Equation 8) $$c_{i}=\frac{1}{\left|c_{i}\right|}\sum_{xÎc_{i}}x$$

f. Duplicate steps (d) and (e) till the locations of the group centers stop changing.

Therefore, the long-term monitoring data (hourly series from 2000 to 2021) from the seven solar radiation observation points in Kyushu (Fukuoka, Oita, Saga, Nagasaki, Kumamoto, Miyazaki, Kagoshima, which are evenly distributed and almost cover the entire region, Figure 8B) are used as input data for extracting solar radiation features. The dimensionless feature profiles are trained and generated using a 24-hour time window.

#### Feature transfer

The dimensionless general parameters (including sunshine duration, cloud cover, and weather conditions) have a strong correlation with solar radiation.^41^ Some parameters, like sunshine duration, can be obtained over long periods at most observation stations. Therefore, by leveraging this correlation, feature transfer can be transformed into pattern recognition and classification tasks. The input parameters are time series of general meteorological data, and the output is the identified feature profiles.

1D Convolutional Neural Network (CNN) is highly effective in extracting features of interest from shorter segments (with fixed length, i.e., kernel size) of the overall dataset, especially when the location of these features within the data segment is not highly correlated.^48^ The network architecture comprises convolutional, batch normalization, rectified linear unit activation, and max-pooling layers (Figure 8E). The convolutional layer function as local feature extractors, performing kernel operations over the spatial domain of the input to capture representative patterns in both width and height dimensions. The feature maps of both the one-dimensional and two-dimensional convolutional layers are derived by employing convolution operations between the input signal and their respective kernel, as formulated in Equations 9 and 10.

(Equation 9) c(i)=x(i)∗w(i)=∑x(j)×w(i−j)

(Equation 10) c(n,m)=x(n,m)∗w(n,m)=∑∑x(s,t)×w(n−s,m−t)

The convolved features maps are then processed by a batch normalization layer that normalizes the preceding activations to stabilize the learning process. The corresponding output features can be represented as:

(Equation 11) $$c_{n}^{l}=\sigma \left(\text{BN}\left(b_{n}^{l}+\sum z_{n}^{l-1}*w_{\text{nm}}^{l}\right)\right)$$

where *w*_*nm*_^*l*^ represents the convolutional kernel between n-th and m-th export characteristic. *Z*_*n*_^*l*^ and *z*_*m*_^*l-1*^ are *n*-th and *m*-th output feature in layer *l*. Function (σ (•)) denotes the regularized linear unit activation as described by Equation 12:

(Equation 12) $$\sigma \left(x\right)=\left\{ \begin{array}{l} x,\geq 0\\ 0,x<0 \end{array}\right.$$

The pooling layer engages non-linear down subsampling and decreases the resolution of the features. The features generated by the max-pooling layer can be represented by Equation 13.

(Equation 13) $$c_{k}^{l}=\max_{\prime \prime Î\Omega_{k}}c_{p}^{l}$$

where Ω_*k*_ represents the pooling area by index *k*.

Network configuration and training parameters can be found in Table S1. The model is trained using a cross-entropy loss function, and overfitting is mitigated through standard strategies including train–validation data splitting, early stopping based on validation performance, and regularization techniques applied during training.

#### Inverse distance weighting interpolation

Inverse Distance Weighting (IDW) method^49^ is a commonly used spatial interpolation method that estimates values at unobserved locations by assigning weights based on the distance to surrounding observed points (Figure 8F). The underlying assumption of IDW is that locations closer to the target point exert a stronger influence on their value than those farther away. In this study, IDW is applied to interpolate the reconstructed solar radiation data at unobserved sites after the representative radiation profiles and corresponding hourly values have been determined at observation stations. The closer the sample point is to the unknown point, the greater the weight assigned. The calculation method can be described as follows:

(Equation 14) $$Z\left(x\right)=\frac{\sum_{i=1}^{n}\frac{Z\left(x_{i}\right)}{d{\left(x,x_{i}\right)}^{p}}}{\sum_{i=1}^{n}\frac{1}{d{\left(x,x_{i}\right)}^{p}}}$$

Where *Z(x)* indicates calculated value at the unknown position *x*. And *Z(xi)* donates the predicted or measured value at the known position *x*_*i*_. And term *d(x, x*_*i*_*)* represents the spatial distance between these two positions. The power parameter *p* is set to 2 in this study.

#### Dataset collection

In order to verify the effectiveness of the proposed method and ensure fairness, the dataset used in this study consists of publicly available data collected and accumulated over the long term by the Japan Meteorological Agency (JMA). Basic information about the meteorological sites can be found in Table S2. This includes seven official meteorological stations (located in Fukuoka, Oita, Saga, Nagasaki, Kumamoto, Miyazaki, and Kagoshima), responsible for collecting data such as solar radiation. Additionally, there are 75 general meteorological recording stations. The dataset covers a period from Jan. 1, 2000, to Dec. 31, 2021, totaling 22 years. It includes hourly observations of various meteorological parameters throughout this time span.

The geographical and meteorological parameters adopted in this work are listed in Table 2, the selection of parameters is based on our previous studies^28^^,^^42^ and the availability of the data:

• Sunshine duration: the period during which solar radiation intensity exceeds 120 W/m^2^, observed in hours (h), was employed as one of the model inputs. Preliminary analysis revealed a strong correlation and temporal consistency between sunshine duration and global solar radiation.
• Time and geographic information: time and geographical information were used to calculate the extraterrestrial solar radiation profile for the observation station.

A comprehensive quality control procedure was applied to all datasets. The hourly solar radiation data records were validated using the criterion (0≤*I*≤0.9·*I*_*0*_) to ensure physical plausibility.

The missing observations and data were reconstructed through linear interpolation within continuous time sequences, minimizing potential biases in the analytical results.

### Quantification and statistical analysis

Statistical analyses were performed using MATLAB (MathWorks, R2024a). To quantify the spatial variability of solar radiation across Kyushu Island, pairwise comparisons of the mean values and standard deviations among seven observational sites were conducted. In addition to independent two-sample t-tests for detecting differences, Pearson correlation coefficients were calculated to evaluate the similarity between sites. The results show that solar radiation characteristics across all sites exhibit strong and statistically significant correlations (all p < 0.001), indicating high spatial coherence across Kyushu Island. For model validation, predicted and observed solar radiation at Saga and Oita stations were compared under five weather conditions, including clear days, overcast days, clear-to-overcast days, overcast-to-clear days, and clear-interval-overcast days. For each weather category, paired t-tests were conducted to assess statistical differences between predicted and measured values. All comparisons showed statistical significance (all p < 0.001). In addition, model performance was evaluated using standard metrics such as RMSE, MSE, and R^2^ to quantify prediction accuracy.

## Supplemental information

Document S1. Figures S1 and S2 and Tables S1 and S2

Data S1. Excel file containing the datasets used in this study after quality control
