# Supplementary material for: Optimizing solar energy planning: A spatial zoning method based on characterized dimensionless radiation profiles
Source: iScience. 2026 Mar 31;29(5):115545. doi: 10.1016/j.isci.2026.115545 (PMC13092865; doi:10.1016/j.isci.2026.115545)
Supplement: Document S1. Figures S1 and S2 and Tables S1 and S2 [file mmc1.pdf]

iScience, Volume *29*

Supplemental information

Optimizing solar energy planning: A spatial zoning method based on characterized dimensionless radiation profiles

Yafei Wang, You Li, Chenhao Ren, and Haolin Yang

Document S1

S.1 Experiments

Table S1. Network configuration and training parameters of the proposed CNN model

Category	Parameter	Value
Model structure	Network type	Convolutional Neural Network (CNN)
Input setting	Input length	24
Convolution layer	Filter size	3
	Stride	1
Pooling layer	Pooling type	Max pooling
	Pool size	5
	Pooling stride	1
Activation function	ReLU	-
Optimizer	Stochastic Gradient Descent with Momentum (SGDM)	-
Initial learning rate	0.01	-
Maximum epochs	300	-
Mini-batch size	15	-

S.2 Basic information on meteorological stations

The meteorological parameters used in this study were collected by the Japan Meteorological Agency. Basic information about the meteorological sites can be found in **Table S2**. Descriptions of the meteorological stations can be found in https://www.jma.go.jp/jma/en/Activities/surf/surf.html.

Table S2 Basic information of meteorological stations in Kyushu.

Observation No.	Station name	Latitude (°N)	Longitude (°E)	Type	Collected data in this research
82046	Munakata	33.80833	130.5383	AMeDAS	*S*
82056	Yahata	33.85167	130.7433	AMeDAS	*S*
82101	Yukuhashi	33.71333	130.975	AMeDAS	*S*
82136	Izuka	33.65167	130.6933	Official meteorological station	*S*
82171	Maebaru	33.56	130.19	AMeDAS	*S*
82182	Fukuoka	33.58167	130.375	Official meteorological station	*S, I*
82191	Daizaifu	33.49667	130.49	AMeDAS	*S*
82206	Soeda	33.55833	130.855	AMeDAS	*S*
82261	Asakura	33.40667	130.695	AMeDAS	*S*
82306	Kurume	33.30333	130.4933	AMeDAS	*S*
82317	Kurogi	33.225	130.645	AMeDAS	*S*
82361	Omuta	33.00667	130.4667	AMeDAS	*S*
83021	Kunimi	33.67833	131.575	AMeDAS	*S*
83061	Bungotakada	33.57	131.4333	AMeDAS	*S*
83106	Innai	33.42	131.3167	AMeDAS	*S*
83121	Kitsuki	33.41667	131.5967	AMeDAS	*S*
83137	Hita	33.32167	130.9283	Official meteorological station	*S*
83191	Kusu	33.29167	131.155	AMeDAS	*S*
83201	Yufuin	33.25333	131.3467	AMeDAS	*S*
83216	Oita	33.235	131.6183	Official meteorological station	*S, I*
83341	Inukai	33.065	131.6317	AMeDAS	*S*
83371	Taketa	32.97333	131.3983	AMeDAS	*S*
83401	Saiki	32.95	131.9017	AMeDAS	*S*
83431	Ume	32.845	131.675	AMeDAS	*S*
83476	Kamae	32.795	131.9233	AMeDAS	*S*
84121	Ashibe	33.8	129.7217	AMeDAS	*S*

84171	Hirado	33.36	129.55	Official meteorological station	*S*
84341	Arikawa	32.98167	129.1183	AMeDAS	*S*
84496	Nagasaki	32.73333	129.8667	Official meteorological station	*S, I*
84536	Fukue	32.69333	128.8267	Official meteorological station	*S*
84561	Kuchinotsu	32.61167	130.1933	AMeDAS	*S*
85116	Imari	33.26667	129.8783	AMeDAS	*S*
85142	Saga	33.265	130.305	Official meteorological station	*S, I*
85161	Ureshino	33.11667	129.995	AMeDAS	*S*
85166	Shiroishi	33.18333	130.1483	AMeDAS	*S*
86006	Kahoku	33.115	130.6917	AMeDAS	*S*
86066	Minamioguni	33.10333	131.0667	AMeDAS	*S*
86086	Taimei	32.915	130.5117	AMeDAS	*S*
86101	Kikuchi	32.945	130.7817	AMeDAS	*S*
86111	Aso Otohime	32.94667	131.04	AMeDAS	*S*
86141	Kumamoto	32.81333	130.7067	Official meteorological station	*S, I*
86161	Takamori	32.82167	131.125	AMeDAS	*S*
86216	Misumi	32.61167	130.4783	AMeDAS	*S*
86236	Kousa	32.67167	130.8167	AMeDAS	*S*
86271	Matsushima	32.515	130.4467	AMeDAS	*S*
86316	Hondo	32.46833	130.18	AMeDAS	*S*
86336	Yatsushiro	32.47333	130.6067	AMeDAS	*S*
86451	Minamata	32.205	130.4067	AMeDAS	*S*
86467	Hitoyoshi	32.21667	130.755	Official meteorological station	*S*
86477	Ue	32.225	130.905	AMeDAS	*S*
86491	Ushibuka	32.19667	130.0267	Official meteorological station	*S*
87041	Takachiho	32.71167	131.29	AMeDAS	*S*
87066	Furue	32.71167	131.82	AMeDAS	*S*
87071	Kuraoka	32.64333	131.1567	AMeDAS	*S*

87141	Nobeoka	32.58167	131.6567	Official meteorological station	*S*
87181	Hyuga	32.40833	131.6	AMeDAS	*S*
87206	Mikado	32.385	131.3317	AMeDAS	*S*
87231	Nishimera	32.24	131.1517	AMeDAS	*S*
87293	Takanabe	32.12333	131.475	AMeDAS	*S*
87301	Kakutou	32.04667	130.81	AMeDAS	*S*
87331	Saito	32.06333	131.4133	AMeDAS	*S*
87352	Kobayashi	32	130.9533	AMeDAS	*S*
87376	Miyazaki	31.93833	131.4133	Official meteorological station	*S, I*
87426	Miyakonojo	31.73	131.0817	Official meteorological station	*S*
87501	Kushima	31.465	131.22	AMeDAS	*S*
88061	Akune	32.02667	130.2	Official meteorological station	*S*
88081	Okuchi	32.04667	130.6267	AMeDAS	*S*
88131	Nakakoshiki	31.835	129.8667	AMeDAS	*S*
88151	Sendai	31.83333	130.315	AMeDAS	*S*
88261	Higashiichiki	31.66833	130.3283	AMeDAS	*S*
88286	Makinohara	31.66167	130.8433	AMeDAS	*S*
88317	Kagoshima	31.555	130.5467	Official meteorological station	*S, I*
88331	Kihoku	31.58833	130.855	AMeDAS	*S*
88371	Kaseda	31.415	130.325	AMeDAS	*S*
88406	Shibushi	31.47833	131.095	AMeDAS	*S*
88432	Kiire	31.38667	130.54	AMeDAS	*S*
88442	Kanoya	31.39167	130.8633	AMeDAS	*S*
88447	Kimotsukimaeda	31.34	130.9383	AMeDAS	*S*
88466	Macurasaki	31.27167	130.2917	Official meteorological station	*S*
88486	Ibusuki	31.25	130.6367	AMeDAS	*S*
88506	Uchinoura	31.27667	131.055	AMeDAS	*S*
88536	Tashiro	31.19833	130.8433	AMeDAS	*S*

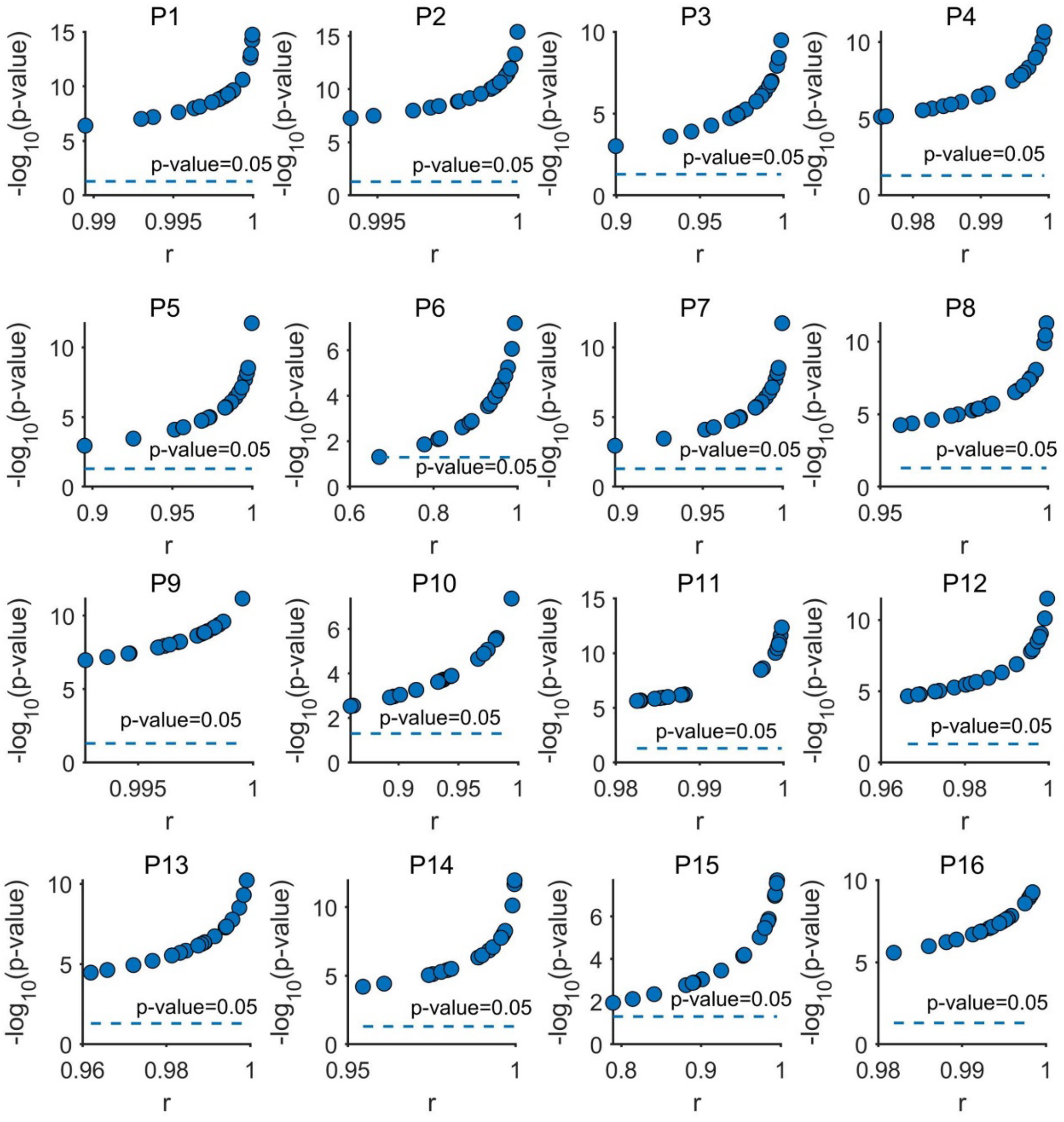

Figure S1. Pairwise correlation and significance testing among the sixteen representative feature types. (Note: Data between 0:00 and 7:00, as well as between 17:00 and 23:00, has been excluded for correlation analysis to prevent an overestimation of model accuracy at low solar altitude angles. These changes do not affect the conclusions)

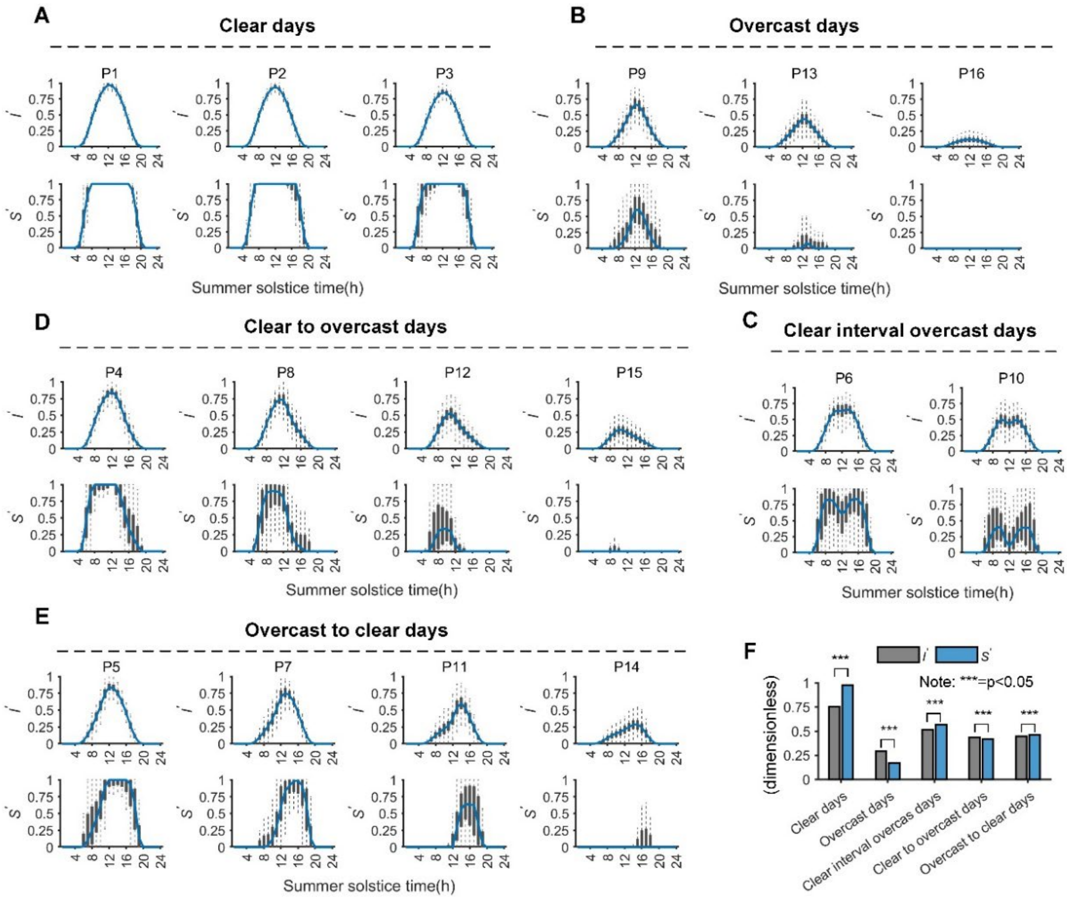

Figure S2. The experimental results conducted in Tokyo (35°69’ N,139°75’E) using the proposed method described in this paper.
